# Functionality of the mineralized cartilage of shark vertebral centra

**DOI:** 10.1063/4.0000854

**Published:** 2025-10-27

**Authors:** Stuart R. Stock, Jason T Parker, Jackson - Comes, Jong - Seto, Michelle S. Passerotti, Lisa J. Natanson, Dilworth Y. Parkinson, Jun S. Park

**Affiliations:** 1 Northwestern University, Chicago, IL, USA; 2 Advanced Light Source, Lawrence Berkeley National Laboratory, Berkeley, CA, USA; 3 Arizona State University, Tempe, AZ, USA; 4 National Marine Fisheries Service, NOAA, Narragansett, RI, USA; 5 Independent Scientist, Homer, AK, USA; 6 Advanced Photon Source, Argonne National Laboratory, Lemont, IL, USA

## Abstract

Although it is not neural tissue per se, the vertebral column is essential to vertebrate animals’ nervous systems. Shark vertebral bodies (centra) consist of mineralized cartilage containing a bioapatite not too different from that in bone. The shark centra possess remarkable resistance to millions of cycles of in vivo strains exceeding 4-8%. These strains are enormous for a mineralized tissue, and there is no repair mechanism for fatigue damage, unlike bone which remodels. It appears that the shark centra evolved to achieve this performance through a hierarchy of structures spanning dimensions from centimeters to nanometers. This talk presents recent x-ray results which have illuminated the structural variations contributing to this functionality. At the 100 μm scale, 3D mapping with energy dispersive diffraction reveals variation of cartilage fiber orientation and bioapatite crystallographic texture between the different structural zones of the centra. At the 1 μm scale, synchrotron microComputed Tomography (microCT) demonstrates that the centra consist of a continuous network of closely-spaced, mineralized trabeculae separated by an interconnected volume of cellular spaces. In situ loading and synchrotron microCT imaging shows that large applied strains are accommodated by rotation/deflection of the trabeculae without a significant increase in stress. Ongoing work includes quantification of cartilage fiber orientations at the level of individual trabeculae.

